# Immunopathogenesis of Pediatric Localized Scleroderma

**DOI:** 10.3389/fimmu.2019.00908

**Published:** 2019-04-30

**Authors:** Kathryn S. Torok, Suzanne C. Li, Heidi M. Jacobe, Sarah F. Taber, Anne M. Stevens, Francesco Zulian, Theresa T. Lu

**Affiliations:** ^1^Division of Pediatric Rheumatology, Department of Pediatrics, Childrens's Hospital of Pittsburgh, University of Pittsburgh, Pittsburgh, PA, United States; ^2^Division of Pediatric Rheumatology, Department of Pediatrics, Hackensack University Medical Center, Hackensack, NJ, United States; ^3^Hackensack Meridian School of Medicine at Seton Hall University, Clifton, NJ, United States; ^4^Department of Dermatology, UT Southwestern Medical Center, Dallas, TX, United States; ^5^Division of Pediatric Rheumatology, Department of Rheumatology, Hospital for Special Surgery, New York, NY, United States; ^6^Department of Pediatrics, Weill Cornell Medicine, New York, NY, United States; ^7^Division of Pediatric Rheumatology, Department of Pediatrics, University of Washington, Seattle, WA, United States; ^8^Seattle Children's Research Institute, University of Washington, Seattle, WA, United States; ^9^Pediatric Rheumatology Unit, Department of Woman's and Child's Health, University of Padua, Padua, Italy; ^10^HSS Research Institute, Hospital for Special Surgery, New York, NY, United States; ^11^Department of Microbiology and Immunology, Weill Cornell Medicine, New York, NY, United States

**Keywords:** localized scleroderma, morphea, pediatric rheumatology, immunophenotype, disease etiology, autoimmune disease, skin, fibrosis

## Abstract

Localized scleroderma (LS) is a complex disease characterized by a mixture of inflammation and fibrosis of the skin that, especially in the pediatric population, also affects extracutaneous tissues ranging from muscle to the central nervous system. Although developmental origins have been hypothesized, evidence points to LS as a systemic autoimmune disorder, as there is a strong correlation to family history of autoimmune disease, the presence of shared HLA types with rheumatoid arthritis, high frequency of auto-antibodies, and elevated circulating chemokines and cytokines associated with T-helper cell, IFNγ, and other inflammatory pathways. This inflammatory phenotype of the peripheral blood is reflected in the skin via microarray, RNA Sequencing and tissue staining. Research is underway to identify the key players in the pathogenesis of LS, but close approximation of inflammatory lymphocytic and macrophage infiltrate with collagen and fibroblasts deposition supports the notion that LS is a disease of inflammatory driven fibrosis. The immune system is dynamic and undergoes changes during childhood, and we speculate on how the unique features of the immune system in childhood could potentially contribute to some of the differences in LS between children and adults. Interestingly, the immune phenotype in pediatric LS resembles to some extent the healthy adult cellular phenotype, possibly supporting accelerated maturation of the immune system in LS. We discuss future directions in better understanding the pathophysiology of and how to better treat pediatric LS.

## Disease Manifestations and Unique Features of Localized Scleroderma (LS) In Childhood

Localized scleroderma (LS) is the most common form of pediatric scleroderma, a disease whose histologic pathology involves inflammation and fibrosis, similar to that of systemic sclerosis (SSc), although the clinical phenotypes are markedly different. Overall, the annual incidence in the US of LS collectively in adults and children is slighter higher than for systemic sclerosis (SSc) (2.7 vs. 1.9 per 100,000, respectively) (1, 2). A much larger difference is found for childhood onset of these diseases, however, with 34% of LS beginning in childhood (by age 18 years), compared to < < 5% of SSc (by age 16 years) (2, 3).

The mean age of pediatric LS disease onset is 6.4–8.7 years, with the disease more prevalent in Caucasians (4). As is true for other autoimmune disease, females are more commonly affected than males (2.3 4:1 ratio) (4). This female preponderance is consistent over different ethnicities, as clinical centers with more homogeneous racial populations, such as Mexico (5), have reported similar ratios of female:male patients.

LS can present in several different patterns (subtypes) depending on depth and distribution of lesions, including circumscribed morphea (plaque lesions), linear scleroderma of the trunk/limb or head (band-like lesion), generalized morphea (multiple plaque lesions), pansclerotic morphea, or a combination of two or more of these subtypes (mixed morphea) (6). Besides the obvious difference in age of disease onset, there are several major differences between pediatric and adult onset disease. These include a different subtype predominance, higher frequency of deep tissue and extracutaneous involvement in pediatric disease, and longer disease duration in pediatric disease [reviewed in (4)]. These differences contribute to the higher frequency of serious morbidity in patients with pediatric compared to adult onset disease (4, 7), with morbidity including arthropathy, uveitis, facial hemiatrophy, seizures, and neuropathy (7, 8). Functional impairment has been reported in 30–38% of juvenile LS patients (9–11).

Twenty to 70% of juvenile LS patients have been reported to have extracutaneous involvement, with higher frequencies reported in prospective studies (7, 8, 11–15). The most common type of extracutaneous involvement is musculoskeletal, which includes joint, tendon, muscle, and bone issues. Joint and tendon issues include arthralgia, arthritis, joint contractures, and angulation defects, some of which require corrective surgeries (16). Muscle involvement includes myalgia, myositis, and muscle atrophy (8). Because the disease commonly begins before most children have undergone their major growth spurt, children are at risk for undergrowth of the affected side, which can lead to limited physical function, pain, and major disfigurement. Growth impairment is common, with studies reporting facial hemiatrophy in half the patients with linear scleroderma of the head, deformity from tissue atrophy and/or muscle bulk reduction in half, and a bone length difference in 15–18% of patients (15–17).

Central nervous system (CNS) involvement is a less common but notable extracutaneous manifestation. The overall frequency of CNS involvement in juvenile LS is ~5% but in patients with linear scleroderma of the head (LSh), it ranges between 44% (18) and 50% (19). Seizures, in particular, partial complex seizures, are the most common neurological symptom, followed by headache, hemiparesis, cranial nerve palsy, optic neuritis; and less commonly, neuropsychiatric disorders, deterioration of intelligence, and/or ischemic stroke (8). Temporal relationship between onset of the neurological symptoms and skin lesions is variable, with the majority of the patients having preceding scalp and facial lesions before CNS presentation, though approximately one-quarter of the cases can present with neurological manifestations (20) (21). Radiological and cerebrospinal fluid (CSF) laboratory findings in LSh patients further support that the disease affects the CNS, likely in an autoimmune manner. When brain imaging is performed in symptomatic patients, abnormalities, such as cortical and subcortical white matter lesions, atrophy, and calcinosis are common, with 34 of the 54 reported patients in one review (63%) found to have multiple or diffuse brain lesions on magnetic resonance imaging (MRI) (20). These brain lesions seem to be more epileptogenic than other autoimmune diseases, such as multiple sclerosis (20). Furthermore, analysis of CSF obtained via lumbar puncture reveals findings consistent with an inflammatory process in some LSh patients demonstrating oligoclonal bands, elevated IgG levels, and autoantibodies (22–24). Further evidence supporting CNS inflammation includes histologic findings of LSh brain biopsies, which demonstrate the same changes as seen in skin: chronic perivascular lymphocytic inflammation with some vessels showing intimal thickening and hyalinization (25).

## Potential Pathogenic Etiologies

### Genetics

Familial history of disease, either immediate or remote, is common for those with autoimmune diseases. For LS patients, 10–30% of patients reported having a family history of autoimmune disease, such as lupus and arthritis (26–30). Ten percent of LS patients have concurrent autoimmune diseases; in children, the most commonly identified diseases are vitiligo, alopecia areata, and juvenile rheumatoid arthritis (26).

Few studies have examined HLA associations in LS. The largest to date was performed using participants from the Morphea in Adults and Children Cohort, which includes about 1/3 childhood-onset LS. In this case control study, HLA Class II genotyping and SSCP typing of HLA A, B, and C alleles was performed and associations between HLA-Class I and II alleles and LS as well as its subphenotypes was determined. Notably, there was only one common allele with adult SSc, DRB^*^04:04, implying LS and SSc are immunogenetically distinct. In contrast, the strongest associations were with DRB1^*^04:04 and HLA-B^*^37 (31). DRB1^*^04:04 is also strongly associated with risk for rheumatoid arthritis. Interestingly, population based studies examining the autoimmune profile of RA have identified increased risk (SIR) of LS in patients with RA. Conversely, studies have indicated there may be increased risk of RA in LS patients. Taken together, this implies that there may be common genetic susceptibility in LS and RA as well as other autoimmune disorders (27, 32). The strong association of LS with specific class-I HLA alleles supports the role of CD8 or natural killer cell associated immune responses in the pathogenesis of LS and implicate loss of tolerance to an unknown self-antigen (31).

### Developmental Etiology

**Figure 1:**
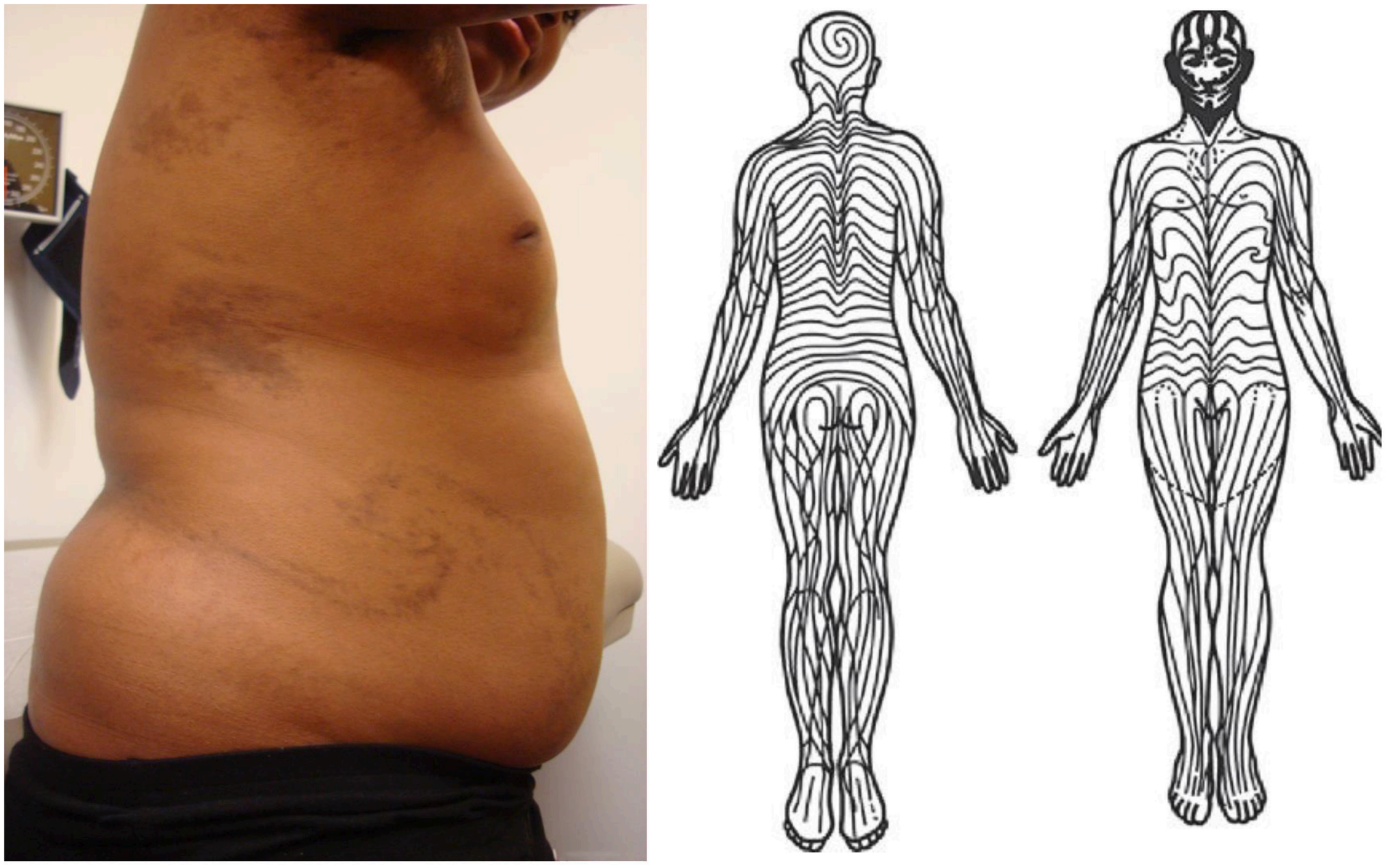
(Left) Swirling lines of Blaschko on patient's right trunk with pediatric-onset localized scleroderma. Note several other patches of morphea on trunk and axilla. She also had linear bands of fibrosis traveling down posterior aspects of bilateral arms and right greater than left leg following lines of Blaschko. Written informed consent was obtained for the clinical photograph. (Right) Diagram of lines of Blaschko. Without modification from Tenea (33).

Several studies have confirmed that localized scleroderma follows the distribution pattern of Blaschko lines, invisible patterns in the skin, distinct from dermatomes, which is a normal embryonic pattern (Figure 1) (34, 35). This includes a retrospective chart review of 65 children with linear scleroderma, plotting skin lesions on standardized head and body charts and then comparing these clinical diagrams to a computer-assisted comparison, which demonstrated excellent correlation (35). Some dermatologic diseases which distribute along lines are believed to be evidence of genomic mosaicism, caused by a somatic mutation during embryogenesis that gives rise to an individual with two genetically distinct populations of cells (36). Whole exome sequencing has been used to prove that affected tissue from other conditions that follow Blaschko lines, including some types of epidermal nevi, contains somatic mutations not present in germline tissue from the same patient (37). Though these findings suggest that localized scleroderma is a form of cutaneous mosaicism, this theory has not yet been confirmed on a molecular basis.

A neuroectodermal origin has been postulated for CNS disease in juvenile LS. Given that face and brain parenchyma tissues derive from a common progenitor in the ectodermal site of the neural tube, an early mutation in the rostral area might cause both cerebral dysgenesis and progressive facial hemiatrophy (38). In support of this hypothesis are the coexistence of ipsilateral cutaneous and neurological lesions and reports of Sturg-Weber syndrome-like intracranial lesions and multiple hamartomas in patients with linear scleroderma of the face (39, 40).

### Immune Etiology

#### LS as an Autoimmune Disease

Takehara and colleagues were among the first to recognize and publish the summary of immunologic findings to support localized scleroderma as an autoimmune disorder with serological supportive evidence being the presence of autoantibodies (autoAb), elevated circulating cytokines, their soluble receptors and soluble cell adhesion molecules (41). Further, examination of the histopathology of LS lesions shows close approximation of immune cell infiltrates with sclerosis in inflammatory lesions, further supporting the interplay between immune dysregulation and increased extracellular matrix components including type I collagen in LS (42). Specifically in regards to LSh, oligoclonal bands and elevated IgG in the CSF have been documented, further supporting an autoimmune phenomenon. Additionally, clinical response of skin, musculoskeletal, and neurological manifestation of LS to immunosuppressive agents, such as corticosteroids and methotrexate, further provides support for the autoimmune hypothesis (43–46). Here, we provide an updated summary of the literature that builds upon this foundation, summarize immune signature grouping or speculations of findings, as well as future directions to give a better understanding of the LS immunophenotype.

#### Histopathologic Evidence for Immune Involvement

In a cross-sectional study of 83 patients with localized scleroderma in which 101 biopsy specimens were examined the authors found that the microanatomical location and degree of inflammation and sclerosis were associated with the stage of evolution of the lesion as well as clinical disease manifestations. The authors categorized sclerosis patterns as top heavy (confined to the papillary dermis), bottom heavy (confined to the reticular dermis and beyond), and throughout (extending from the papillary dermis to subcutis and beyond). They also quantified the degree and location of inflammation and cell types present. Important observations included that all patterns of sclerosis (top, bottom, and throughout) were present in circumscribed, generalized, and linear subtypes. Interestingly, regardless of subtype the bottom heavy or throughout pattern of sclerosis was associated with increased risk of pain and functional limitation, implying that both the location of a lesion on the skin as well as the microanatomical location of the pathology are important in determining those at risk for more serious sequelae. In terms of inflammatory cell types, lymphocytes predominated with plasma cells as second most common. Surprisingly eosinophils were present in 21% of specimens. The microanatomical location of inflammation closely mirrored that of the pattern of sclerosis present in an individual specimen. In other words, sclerosis occurred in areas that were enriched in inflammatory cell infiltrate. This implies a link between inflammation and activation of fibroblasts in the surrounding dermis and subcutis. Taken together, these results support the use of microanatomical location of inflammation and sclerosis in assessing risk of pain and functional limitations in localized scleroderma as well as further linking immune dysregulation as a driver of sclerosis (42).

In addition to fibroblasts, the immune cells could potentially activate skin structures. The location of the inflammatory cell infiltrate in skin is typically in a perivascular, peri-eccrine and peri-neural distribution, with the interplay with the neurovascular bundles sometimes termed “lymphocytic neurovasculitis” (47). In CNS disease, chronic perivascular lymphocytic infiltrate with intimal thickening and hyalinization of the vessels are also found when brain biopsies are obtained (23–25). T lymphocytes were also identified in a brain biopsy (48). Together, the histopathology in multiple organs in LS further suggest an autoimmune phenomenon.

#### Autoantibodies and Clinical Associations in LS

Autoantibodies (autoAb) are commonly observed in individuals with LS which reflects activation of the immune system and auto-reactivity to self-antigens. Although not as specific with regard to organ manifestation or scleroderma subtype as seen in systemic sclerosis (SSc), autoAb may be helpful in associating to disease severity and/or depth of LS. When more classic SSc-associated autoAb, such as anti-centromere and anti-topoisomerase, are tested in LS patients, they occur in 3–18% of the LS subjects (4, 8, 26, 49, 50), none of which had or developed SSc in addition to their LS diagnosis. A recent study of the SSc line immunoassay (LIA) (Euroimmun, Germany) in pediatric LS subjects found similar percentages (centromere 14%, topoisomerase 10%, RNA Polymerase III 12%) and when compared to clinical parameters, correlated to deep tissue involvement, signified by joint contractures, muscle involvement and nerve entrapment (51). Interestingly, none of the SSc-antibodies associated with LS subtype designation (51).

A high proportion of pediatric LS patients are Anti-Nuclear Antibody (ANA) positive, ranging from 30 to 70% when tested by indirect immunofluorescence (4, 11, 26–29, 41, 52, 53). The frequency and clinical utility of autoantibodies in LS has been the subject of numerous studies, with varied results. The presence ANA in several studies corresponded to deeper disease involvement (beyond the subcutis) by associating with features, such as joint contractures, muscle atrophy and extremity shortening, but not disease subtype or age of onset (4, 26–29, 53, 54). A recent longitudinal cohort study supports a positive ANA at LS diagnosis to be predictive of likelihood for recurrence; therefore, ANA is likely promoting autoimmunity in some fashion or reflecting a more auto-reactive state (55). Other auto-antibodies which may be reflecting positive ANA in LS have been reported, most commonly, anti-histone antibody (AHA) and anti–single-stranded DNA antibody (ssDNA Ab). In those tested, a range of 10–50% of LS patients are positive for ssDNA and/or AHA (56–58) with both correlating to severity features, such as deep muscle involvement, joint contractures, and increased number of lesions (41, 57, 59), and is able to track with disease activity status in a subset of patients (60).

When evaluated, rheumatoid factor was present in 16- 29% of patients and associated with arthritis (29, 50). Other markers of immune activation, such as IgG, IgA, and IgM were found to be increased in patients with linear scleroderma, and deep and pansclerotic morphea (29). Elevation of other more typical laboratory parameters tested in connective tissue diseases, such as muscle enzymes, corresponded to deeper tissue involvement. Elevated creatine phosphokinase (CPK) and aldolase were associated with disease parameters including muscle atrophy and extremity shortening in a North American pediatric LS cohort, indicating muscle involvement (11).

#### Cytokine and Cellular Signatures in LS

The exact cellular signature of LS is still being investigated. In both adult and pediatric LS analyses, lymphocytes and their associated cytokine and chemokine populations are observed in both the blood and skin. Flow cytometry studies of the circulating cellular phenotype of LS (pediatric and adult) have shown a predominance of CD4^+^T helper cells along with decreased functional T regulatory cells (61–63). This decrease in T regulatory cells, possibly reflecting a more “permissive state,” was also seen in pediatric SSc without increases in other T cell populations (64). Furthermore, when comparing paired active to inactive PBMC phenotypes in LS, those with active disease states demonstrated much higher populations of IFNγ-expressing T cells (reflecting T_H_1 cells; CD4^+^ IFNγ + T cells) (63). An expanded study utilizing multiparameter mass cytometry by time-of-flight spectrometry (CyTOF) also supported increased IFNγ expression from CD4^+^T cells, as well as NK cell populations, in active LS PBMC samples (65).

T helper cells consist of 3 main types including T_H_1, T_H_2, and T_H_17. These cell types produce distinct interactive cytokine profiles. T_H_1 cells secrete IFN-γ and IL-2 and are stimulated by IL-12, whereas T_H_2 cells produce IL-4, IL-5, IL-6, IL-10, and IL-13 and are activated by IL-4. T_H_17 cells produce IL-17 A/F, IL-21, IL-22 and are propagated by IL-23, IL-6, and IL-1. While elevation of cytokines associated with all three T_H_ lineages have been observed in LS, the pattern of expression is consistent with that of a T_H_1 predominance (Table 1). In peripheral blood, elevation of T_H_1 related cytokines, chemokines, and their receptors include: IL-2 (67, 69, 73), IL-12 (66, 67), TNFα (70), TGFβ (66, 67, 71, 72), MCP-1 (66), IFNγ related proteins, CXCL9 (monokine induced by gamma interferon [MIG]) (67, 76, 77), CXCL10 (Interferon gamma-induced protein 10 [IP-10]) (67, 76) and CXCL11 (Interferon-inducible T-cell alpha chemoattractant [I-TAC]), and IFNγ chemokine receptor (CXCR3). T_H_2 related cytokines IL-4 (69), IL-6 (69, 72), IL-13 (70), and T_H_17 related cytokines, IL-17A (66, 75), and IL-23 (75), were also reported as significantly elevated in peripheral blood of LS patients compared to controls. Peripheral blood levels of the IFNγ-related chemokines CXCL9 and CXCL10 also correlated to disease activity measures, such as clinical scores, the modified Localized scleroderma skin severity index (mLoSSI), and the Physician Global Assessment of disease activity (66, 67, 78) (Table 1), underscoring their potential as serological biomarkers of disease activity.

**Table 1:** Peripheral Blood Cytokine Profiles in LS: Elevated cytokines associate with T helper cell linages and correlate with other inflammatory disease indicators, such as activity scores and clinical laboratory tests.

		**Patient (n) and LS, SSc**	**Subtype**	**Ages median (IQR)mean ± SD**	**Sex (% female)**	**Clinical and serological correlations**
CCL2/MCP-1	Torok et al. (66)^†^	69 Ped LS	8 generalized 41 linear 8 plaque morphea	13.0 (10.0–16.0)	67%	–
CCL3/MIP-1a	O'Brien et al. (67)^†^	87 LS	49 generalized 28 linear 8 plaque morphea	50 ± 20 years (89% adult)	69%	–
CXCL8/IL−8	Ihn et al. (68)	48 LS 20 SSc	16 generalized 22 linear 10 plaque morphea	–	–	–
IL-2	Ihn et al. (69)	48 LS 20 SSc	16 generalized 22 linear 10 plaque morphea	–	–	RF
IL−2R	O'Brien et al. (67)^†^	87 LS	49 generalized 28 linear 8 plaque morphea	50 ± 20 years (89% adult)	69%	LoSDI
IL−4	Ihn et al. (69)	48 LS 20 SSc	16 generalized 22 linear 10 plaque morphea	–	–	AHA
IL−6	Ihn et al. (69)	48 LS 20 SSc	16 generalized 22 linear 10 plaque morphea	–	–	AHA
IL−13	Hasegawa et al. (70)^†^	45 LS	12 generalized 22 linear 11 plaque morphea	27 (range 5–67 years old)	59%	Number of plaque lesions Number of lesions
IL−12	Torok et al. (66)^†^	69 Ped LS	8 generalized 41 linear 8 plaque morphea	13.0 (10.0–16.0)	67%	–
	O'Brien et al. (67)^†^	87 LS	49 generalized 28 linear 8 plaque morphea	50 ± 20 years (89% adult)	69%	LoSDI
TNFα	Hasegawa et al. (70)^†^	45 LS	12 generalized 22 linear 11 plaque morphea	27 (range 5–67 years old)	59%	IgM AHA IgG ssDNA Muscle involvement
TGFβ1	Uziel et al. (71)^†^	55 Ped LS	16 generalized 28 linear 10 plaque morphea	9.2 ± 3.6 years	–	–
TGFβ2	Budzynska-Włodarczyk et al. (72)	17 LS	9 generalized 6 other subtypes	45.3 ± 14.6 years	100%	–
sIL−2r	Uziel et al. (73)	17 Ped LS	–	8.1 years	–	–
sIL−6r	Nagaoka et al. (74)	45 LS 20 SSc	12 generalized 22 linear 11 plaque	–	–	IgM RF Number of lesions Number of body areas
	Budzynska-Włodarczyk et al. (72)	17 LS	9 generalized 6 other subtypes	45.3 ± 14.6 years	100%	ESR
	Nagaoka et al, (74)	45 LS 20 SSc	–	–	–	IgM AHA RF Number of lesions Number of body areas
IL−23	Danczak-Pazdrowska et al. (75)^*^	41 LS	14 generalized 7 linear 20 plaque	43.7 ± 17.5 years	53%	mLoSSI in plaque patients Disease duration in all subtypes
IL−17A	Danczak-Pazdrowska et al. (75)^*^	41 LS	14 generalized 7 linear 20 plaque	43.7 ± 17.5 years	53%	mLoSSI in plaque patients Disease duration in all subtypes
	Torok et al. (66)^†^	69 Ped LS	8 generalized 41 linear 8 plaque morphea	13.0 (10.0–16.0)	67%	–
IL−1	Danczak-Pazdrowska et al. (75)	41 LS	14 generalized 7 linear 20 plaque	44 ± 18 years	50%	–
CXCL9./MIG	O'Brien et al. (67)^*^^†^	87 LS	49 generalized 28 linear 8 plaque morphea	50 ± 20 years (89% adult)	69%	mLoSSI LoSDI
	Mertens et al. (76)	80 LS	16 generalized 30 linear 8 plaque morphea	−80% adult	59%	mLoSSI
CXCL10./IP-10	O'Brien et al. (67)	87	49 generalized 28 linear 8 plaque morphea	50 ± 20 years (89% adult)	69%	LoSDI
	Mertens et al. (76)	80 LS	16 generalized 30 linear 8 plaque morphea	−80% adult	59%	mLoSSI
	Magee et al. (77)^*^^†^	69 Ped LS	8 generalized 40 linear 5 plaque morphea	12.5 (10.0–16.0)	46%	PGA-A mLoSSI
sgp130	Nagaoka et al, (74)	45 LS 20 SSc	–	–	–	IgG Number of lesions Number of body areas

* Also demonstrated in skin

† Includes pediatric patients

CCL2/MCP-1, Monocyte chemoattractant protein-1;

CCL3/MIP-1a, macrophage inflammatory protein 1 alpha;

CXCL8/IL-8, interleukin 8;

IL-2, interleukin 2;

IL-2R, interleukin 2 receptor;

IL-4, interleukin 4;

IL-6, interleukin 6;

IL-13, interleukin 13;

IL-12, interleukin 12;

TNFα, Tumor necrosis factor alpha;

TGFβ1, Transforming growth factor beta 1;

TGFβ2, Transforming growth factor beta 2;

sIL-2r, soluble interleukin 2 receptor;

sIL-6r, soluble interleukin 6 receptor;

IL-23, interleukin 23;

IL-17A, interleukin 17A;

IL-1, interleukin 1;

CXCL9/MIG, Chemokine (C-X-C motif) ligand 9/Monokine induced by gamma interferon;

CXCL10/IP-10, C-X-C motif chemokine 10/Interferon gamma-induced protein 10;

Sgp130, soluble gp130;

AHA, anti-histone antibody;

ANA, anti-nuclear antibody;

ESR, Erythrocyte sedimentation rate (sed rate);

IgG, Immunoglobulin G;

IgM, Immunoglobulin M;

LoSDI, Localized Scleroderma Damage Index;

mLoSSI, modified LS Skin Severity Index;

RF, Rheumatoid factor;

ssDNA, single stranded DNA antibody.

**Figure 2:**
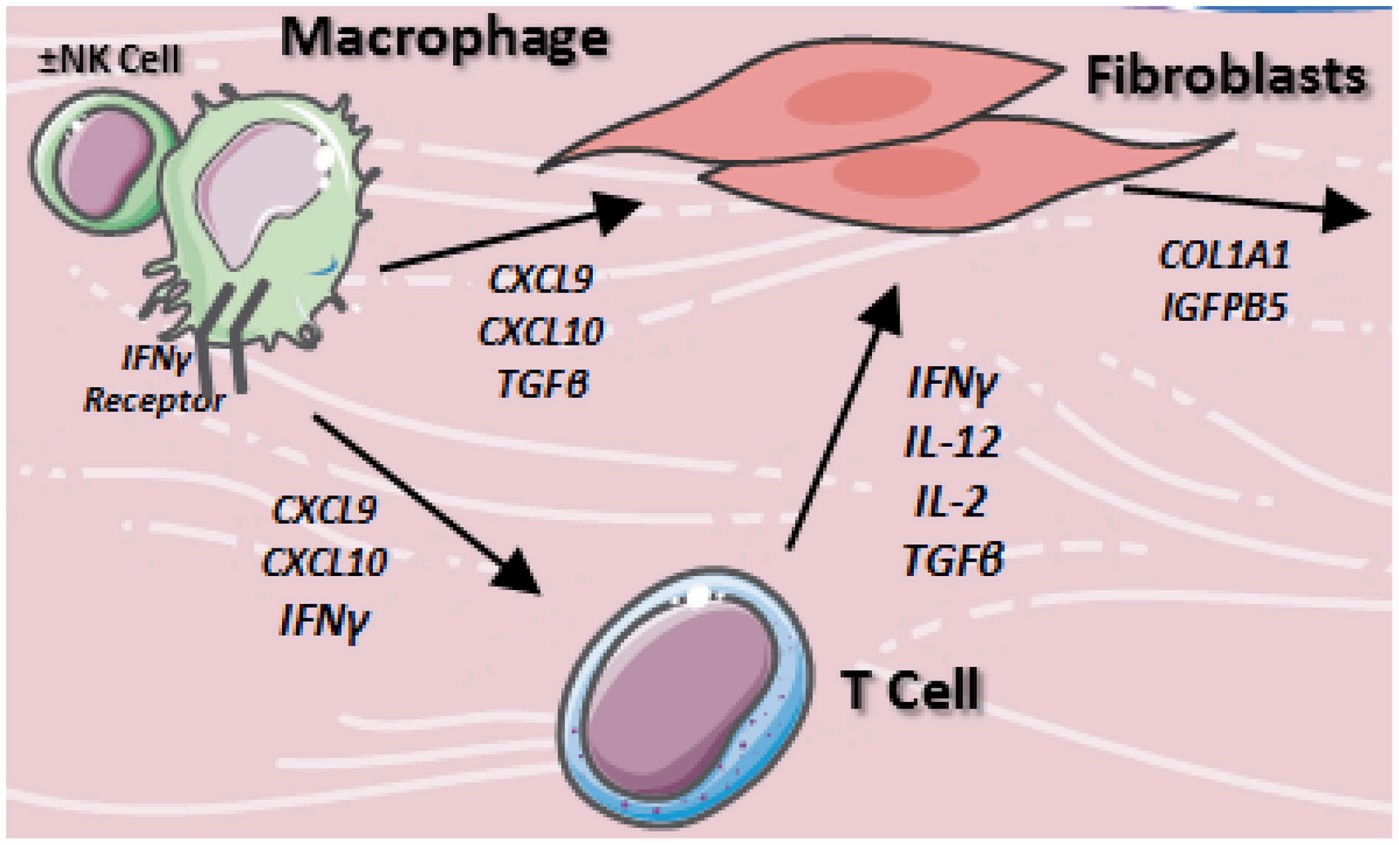
Proposed cellular interactions of macrophages, fibroblasts, and T cells in localized scleroderma. Resident macrophages stimulate T cells and fibroblasts via TH1/IFNγ associated cytokine network to produce inflammation and collagen accumulation in the skin.

In LS skin, T_H_1/IFNγ related chemokines CXCL9 (67) and CXCL10 (77) were increased, while T_H_17 related cytokines IL-23 (75) and IL-17A (75) were decreased compared to healthy control skin. Both CXCL9 and CXCL10 were found to be present in the perivascular lymphocytic infiltrate of the papillary and reticular dermis (67, 77). Additionally, CXCL9 was found to stain in close approximation to both CD4^+^T_H_ cells and macrophages (67), suggesting potential interaction between lymphocytes and macrophages utilizing IFNγ chemokine signaling (Figure 2). Overall, this may synergistically promote fibroblasts to increase collagen expression in LS, eventually causing increased collagen deposition, fibrosis, and a shift toward a later T_H_2 profile.

In summary, in LS there is a T_H_1/IFNγ signature prevalent in the active or initial inflammatory stage of the disease and likely a more fibrotic T_H_2 signature follows in the collagenous stage of LS, which more closely resembles long term SSc disease profiles. These two states of inflammatory and fibrotic disease have unique profiles that reflect the clinical perception of disease. In pediatric patients, CXCL9, CXCL10, CXCL11, MIP-3β, IL-9, IL-2, and CCL-1 were elevated in the active state compared to the inactive state of disease (77, 78) as indicated by these cytokine profiles of the blood and skin. The exact relationship of the circulating cellular and cytokine/chemokine players to skin pathophysiology is still under study. One study focused on skin expression of select transcripts, and found increased mRNA levels of CCR7 and CCL5/RANTES in LS lesions (79). This supported the idea that lymphocytes are recruited from the blood to lesional skin. Investigation into skin-homing T cell profiles are underway in LS, with preliminary data showing that both skin homing CD8^+^(Tc)CCR10^+^ and CD4^+^(T_H_)CCR10^+^ T cells subsets produce inflammatory cytokine populations, including IFNγ, that were significantly increased in the active disease state compared to the inactive disease state (80). In adult SSc, IL-13^+^, and not IFNγ^+^, CD8^+^CCR10^+^ cells appear to be the skin homing cell type that propagates disease (81), emphasizing possible biological differences in scleroderma phenotypes: a more inflammatory, T_H_1/T_C_1 phenotype in LS and a more fibrotic, T_H_2/T_C_2 phenotype in SSc.

#### The LS Transcriptome

Investigation into the transcriptome of scleroderma, specifically SSc, has been successful at classifying samples based on genetic differences that could indicate or predict disease progression and potential future treatment plans. Adult SSc subjects were classified into 4 distinct genetic signatures based on overall microarray expression of the skin (82). These classifications include a *diffuse-proliferation* group composed of diffuse SSc patients, the *inflammatory* group containing diffuse SSc, limited SSc and LS (4 morphea subjects were included in the analyses), the *limited* group composed of limited SSc, and the *normal-like* group, which includes healthy controls along with a few SSc patients (82). All of the LS subjects included fell nicely into the inflammatory group, expressing primarily T-lymphocyte and IFNγ related genes (82), which corresponds to the peripheral blood and protein skin expression discussed above. Recent transcriptional analysis using RNA bulk sequencing of pediatric LS skin showed a distinct subset of patients expressing similar inflammatory genes including interferon-inducible chemokines, such as CXCL9, CXCL10, CXCL11, and IFNγ itself (83). Similar to SSc, subgroupings of LS patients are expected to be present as these inflammatory genes were more highly expressed in LS patients with more inflammatory or active lesions, with associated higher clinical activity scores, mLoSSI and PGA-A (83). This transcriptome classification system in SSc has been used more recently to predict patient response to therapy, such as the inflammatory subset showing better response to mycophenolate mofetil and the fibroproliferative group showing better response to stem cell transplant (84, 85). A methodologically similar classification of LS using immunophenotyping of the transcriptome could help to delineate immunological subtypes and determine therapeutic responses to disease.

#### Speculation: Differences in Immune System Function in Children and Adults

The greater prevalence of extracutaneous manifestations and autoimmunity in children than in adults suggests the possibility of some age-related differences in disease mechanisms that may potentially reflect differences in immune system function between children and adults. The immune system is relatively immature at birth, and many changes occur within the first year of life. Newborn dependency on antibodies from the maternal circulation and immune cells undergo phenotypic changes over the first few months of life including a robust thymic output of T cells that subsides over the first few years (86, 87). However, there are also changes from early childhood to adulthood. For example, in the peripheral blood, CD4 and CD8 T cell counts increase from childhood to adulthood while B cell counts drop (88). At the same time, regulatory T cell (87), monocyte (89) and NK cell (90) counts are higher in infancy or early childhood than in adulthood. Whether these differences in cell population reflect differential generation, trafficking, or survival is currently unclear. T and B cells are continuously trafficking between blood and secondary lymphoid organs, such as spleen and lymph nodes (91), and an increased proportion of B cells in the blood circulation at a young age could reflect relative reductions in B cell entry into lymph nodes, and potentially less exposure to an environment that supports the generation of a robust but also a well-regulated response. Further understanding how differences in immune cell numbers reflect differential immune system function could help to better understand disease.

In addition to differences in cell numbers, immune cells also differ in function in children and adults. Generally, early life is dominated by a more regulatory state that favors healing and repair (92). For innate immunity, NK cell phenotype is more consistent with cytokine producing rather than cytotoxic function early in post-natal life, with more mature phenotype during the first years of life (90). Responses to toll like receptor (TLR) stimulation are reduced during early months to years in Western countries (90, 92). At the same time, production of IL-10, a regulatory cytokine decreases during infancy to below adult levels, and then increases again until adult levels are reached (90). The functional implications of these alterations for different manifestations of scleroderma in childhood and adulthood are unclear, but these differences drive home the point that immunopathogenesis at different times of life may be different.

Childhood is also a time of immune repertoire development for lymphocytes, whereby lymphocytes that are naïve will be exposed to antigen, and antigen- experienced lymphocytes will constitute a larger proportion of the body's lymphocytes (87). Exposure over infancy and childhood through the gut, skin, and respiratory tract to different microbes, food, and the environment is thought to contribute to shaping and increasing the memory T cell compartment and protective antibodies. While the immune repertoire is modulated with intercurrent infections, even in the absence of frank infections, these exposures help to generate memory cells and antibodies that can potentially cross react enough with potential pathogens to protect the child later on (87). In addition to the antigenic exposure, the diversity of an individual's immune repertoire is also shaped by stochastic events. The selection of the exact T and B cells that will dominate in the response to antigen exposure has a large element of randomness, as reflected in the different immune repertoires in twins (93). During this period of high expansion of the immune repertoire, T and B cells that are cross-reactive to self and pathogenic could incidentally be generated.

Given these differences in immune function in children and adults, it is interesting to consider that the biomarkers of disease seen in pediatric LS compared to healthy pediatric controls—the reduced regulatory T cell, increased T_H_1, T_H_2, and T_H_17 cells, and increased innate cell activation—resemble the pattern seen in a healthy adult immune system. We speculate that, while autoreactivity could contribute to pathogenesis, the cell distribution and activity in pediatric LS may actually signal an accelerated development of the immune system. This would be complementary but distinct from “aging” of the immune system and generation of senescent pro-inflammatory cells (94), but an acceleration of normal transition to a more permissive immune system that, perhaps in addition to the increased antigenic exposure during childhood, contributes to pathology in children.

## Future Directions for Research

While treatment strategies effective for most patients have been identified, there is a major need for additional treatment options and strategies. Thirty percent of jLS patients may fail to respond to initial standard immunosuppressive treatment (95), and 15–53% of patients can relapse following treatment (15, 28, 95). Active disease can persist for decades (53, 96). Failure to achieve remission and relapsing disease are both associated with poorer outcome (97).

Identifying optimal treatment strategies for jLS will require comparative effectiveness studies; the feasibility of this approach was demonstrated by a recent pilot study of three standardized methotrexate based regimens (13). Because treatment is focused on controlling inflammation, sensitive monitoring of disease activity is essential for conducting such trials. A recent study identified specific lesion features for tracking disease activity that are likely to improve the sensitivity and specificity of existing clinical measures (14). Future work may lead to development of a weighted clinical activity measure to further improve our ability to identify relative differences in treatment efficacies. The identification of biomarkers that facilitate monitoring activity level and/or help identify response to specific treatments will enable us to work toward personalized medicine for these patients.

Further understanding disease pathophysiology will aid in the development of new therapeutic approaches. Insight into the exact nature of the immune dysfunction and how it contributes to skin fibrosis and the extracutaneous manifestations may help to better target the immune system to treat the disease. Understanding how the unique aspects of the immune system of childhood intersect with the disease process can further provide insight into disease etiology and perhaps teach us how to best reduce the immune dysfunction, or at the very least, repair the damage.

## Author Contributions

KT, SL, HJ, ST, AS, FZ, and TL conceived of and wrote the manuscript.

### Conflict of Interest Statement

The authors declare that the research was conducted in the absence of any commercial or financial relationships that could be construed as a potential conflict of interest.
